# Miller Fisher Syndrome Associated With COVID-19: A Child Case Report and an Up-to-Date Review of the Literature

**DOI:** 10.7759/cureus.35656

**Published:** 2023-03-01

**Authors:** Turan Poyraz

**Affiliations:** 1 Elderly Care, Izmir University of Economics, İzmir, TUR

**Keywords:** sars-cov2, molecular mimicry, miller fisher syndrome, covid-19, anti-gq1b

## Abstract

Miller Fisher Syndrome (MFS) was first recognized by James Collier in 1932 as a clinical triad of ataxia, areflexia, and ophthalmoplegia. In 1956, three cases with this triad were published by Charles Miller Fisher as a limited variant of Guillian-Barré syndrome (GBS), and the disease started to be called by his name. Since the beginning of the severe acute respiratory syndrome coronavirus 2* *(SARS-CoV-2) pandemic, there have been many reports of peripheral and central nervous system involvement. Until December 2022, a total of 23 cases including two children associated with MFS had been reported.

In this article, we present a case of SARS-CoV-2 with classic triad clinical findings, which started with the atypical clinic at an early age. Electrophysiological studies of the case were found to be consistent with sensory axonal polyneuropathy. AntiGQ1b antibody IgG and IgM were negative. The case was spontaneously remitted without IV immunoglobulin (IVIg) or plasma exchange (PE) treatment. A current review of the literature is presented with the smallest pediatric case reported. Based on this case, it was planned to emphasize the targets and highlights in the diagnostic parameters.

## Introduction

Miller Fisher syndrome (MFS) was first described by Collier in 1932 as a variant of Guillain-Barré syndrome (GBS) characterized by ataxia, ophthalmoparesis, and areflexia [1]. In 1956, three cases with this triad were published by Charles Miller Fisher and the disease started to be called by his name [2]. The worldwide incidence of GBS is approximately 1-2 per 100,000 and the MFS variant represents a small subset of these cases (5% of GBS) [3]. Since the beginning of the severe acute respiratory syndrome coronavirus 2 (SARS-CoV-2) pandemic, there have been many reports of peripheral and central nervous system involvement. By December 2022, a total of 24 cases of COVID-19-associated MFS have been reported, including two children [4-7]. Muscle strength is usually maintained and can be accompanied by common ocular findings that can reach the total ophthalmoplegia including fixed dilated pupil. In this article, we report a case of MFS with early electrophysiological findings and completely spontaneous recovery. His blood serum and cerebrospinal fluid (CSF) were negative for antiGQ1b IgM and IgG antibodies but he had the presence of albuminocytologic dissociation (ACD) in CSF.

## Case presentation

A six-year-old male patient had complaints of sore throat, nasal discharge, and fatigue 12 days ago. Following these complaints, double vision, imbalance, and limitation of eye movements were added in a span of few days. The patient was first evaluated by the ophthalmologist and directed to the pediatrician. He was most recently referred to a neurologist. On the sixth day of complaints, the vital signs were stable and in the neurological examination, mental status was found to be normal. He was fully alert and oriented to time, place, and the person with an intact memory. On examination, his visual acuity was 6/6 unaided bilaterally. Color vision tested with Ishihara plates was normal, and visual field testing by confrontation was also normal. He had bilaterally dilated pupils with weakness response to light. Corneal reflexes were normal. The double vision during the early period of his afflictions was absent during the neurological examination. Ocular movements were found to be paretic at various rates in all directions, and the saccadic eye movements could not be maintained, especially in the horizontal gaze. He did not participate in convergence and divergence. Left ptosis was present. Anterior segment examination and fundoscopy were normal on both sides. The remainder of the cranial nerves were intact. Muscle strength in the upper and lower extremities of the patient was complete. His sensory examination was normal. Deep tendon reflexes (DTR) were absent from the upper and lower limbs (biceps, triceps, brachioradialis, patellar, and ankle). He displayed bilaterally significant dysmetria on finger-to-nose and alternating movements testing. The gait was ataxic with predominantly tandem. He also had truncal ataxia. The Romberg test was positive. The plantar responses were flexor. Brain MRI showed that the appearance and intensity of brain parenchyma were normal on a 1.5 T scanning. The ventricular system and cisternal spaces appeared normal. No evidence of intracranial space occupying lesion or obvious vascular anomaly was detected.

It was learned that the patient's first complaints started 12 days ago, and the COVID-19 test, which was given to the child during this period when his parents were infected with COVID-19, turned out to be positive. After the first evaluation, qualitative real‑time reverse transcriptase polymerase chain reaction assay for SARS‑CoV‑2 was repeated via oropharyngeal swab test and it was found that the positivity continued.

Neurophysiology

Nerve conduction studies were done using conventional procedures and a Nihon Kohden, Neuropack S1 MEB-9400 EMG machine. Electrophysiologic assessment (EMG, electromyography and NCS, nerve conduction studies) showed normal motor NCS (CMAP, compound muscle action potentials, NCV, nerve conduction velocity, and distal latency) and reduction of SNAP (sensory nerve action potential) amplitudes in all four extremities. Motor conduction studies were done in the median, ulnar, tibial, and peroneal nerves. F wave latencies could not be obtained from both tibials. Ulnar F waves latency was prolonged. H reflex response recorded from the soleus muscle could not be obtained. Needle insertion could not be performed because EMG could not be tolerated. Based on the present findings, neurotransmission findings compatible with a significant moderate sensory and axonal polyneuropathy were obtained at the bottom.

CSF and other laboratory findings

The cerebrospinal fluid (CSF) analysis showed ACD. Gram stain and CSF culture showed no growth after seven days. He was hospitalized with these results. AntiGQ1b IgG and IgM antibodies detected by ELISA were negative <1700 BTU (<1700 BTU normal). Hemogram was normal. C-reactive protein (CRP) was 24.81 mg/dL and the erythrocyte sedimentation rate (ESR) was 5 mm/h. FreeT3 was found to be 3.89 pmol/L (3.1-6.8), freeT4 was 11.35 pmol/L (12-22), thyroid stimulating hormone (TSH) was 0.394 mlU/L (0.27-4.2), and Vitamin B12 was 551.3 pg/mL (191-663). Other biochemical studies were normal. Herpes simplex virus (HSV) I and II deoxyribonucleic acid (DNA) polymerase chain reaction (PCR) was not detected. Autoimmune and vasculitic panels were negative (Table 1).

**Table 1:** CSF results. CSF, cerebrospinal fluid; AMPA, alpha-amino-3-hydroxy-5-methyl-4-isoxazolepropionic acid receptor; CASPR2, contactine-associated protein-like 2; CMV, cytomegalovirus; EBV, Epstein Barr virus; GABAR, gamma aminobutyric acid receptor; GM, ganglioside monosialic acid; HSV, herpes simplex virus; LGI-1, leucine rich glioma inactivated-1; NMDAR, N-methyl D-aspartate receptor; VZV, varicella zoster virus; WBCs, white blood cells; RBCs, red blood cells; VDRL, venereal disease research laboratory

CSF analysis	Results
Opening pressure (10-18 cm of H_2_O)	10 cm of H_2_O
Color	Clear
Cells (WBC or RBCs)	0
Gram stain and culture	Negative
Glucose (40-70 mg/dL)	50 mg/dL
Protein (15-45 mg/dL)	87 mg/dL
VDRL test	Non-reactive
CMV IgM	Negative
VZV by PCR	Negative
EBV by PCR	Negative
Autoimmune encephalitis panel	
NMDAR	Negative
AMPA-1	Negative
AMPA-2	Negative
CASPR-2	Negative
GABAR-B1/B2	Negative
LGI-1	Negative
Anti-ganglioside panel	
GM1, GM2, GM3	Negative
GD1a, GD1b	Negative
GT1b	Negative
GQ1b	Negative

The patient underwent a care-monitoring session in which the serum Ig-A level was normal but cardiac arrhythmia or insufficiency of the left atrium was not observed. Significant improvement was observed in symptoms after one week. Progression stopped after the second week and significant improvement began in the third week. Recovery at the ataxia was the earliest improvement, while cerebellar regression and ophthalmoparesis remained more moderate but continued. During the weekly neurological follow-up of the patient discharged after the first week, it was observed that there was a near improvement at the end of the third month.

## Discussion

The MFS is an acute polyneuropathy characterized by the triad of ataxia, areflexia, and ophthalmoplegia. The worldwide incidence of GBS is approximately one to two in 100,000, with the MFS variant representing a tiny subset of the cases (one to two in 1,000,000) [3].

In this case, a case with MFS associated with COVID-19 was presented as a result of detailed history and laboratory investigations. The development of neurological symptoms following the subject's infection with SARS-CoV-2 was observed to be similar to that of several other previously documented cases of COVID-19 associated with MFS. While some of the COVID-19-related MFS cases reported so far have severe COVID-19 infection to the point of hospitalization, the vast majority of cases have been reported to have a mild clinical course of infection. In our case, the COVID-19 infection showed a mild clinical course. Again, the fact that patients who test positive for COVID-19 have consistent presentations with MFS despite never having symptomatic COVID-19 highlights that the syndrome can potentially occur in any patient exposed to SARS-CoV-2, regardless of disease severity [4]. Our case is male and is the youngest case reported in the literature [4-7]. This is the third pediatric case reported to be associated with COVID-19 [6-7]. The clinical, laboratory, and demographic data of these 24 cases are summarized in Table 2.

**Table 2:** Study origin, demographics, and GQ1b Ab. *Anti-GD1b:+; N/A, non-available; M, Male; F, Female

Study number	Country	Age (years)	Gender	No. of patient	COVID-19 symptoms	Anti-GQ1b
1	Indian	7	M	1	Available	Negative
2	Saudi Arabia	11	M	1	None	N/A
3	Spain	51	F	1	Available	Negative
4	Italy	50	F	1	Available	Negative
5 (two cases)	Spain	50, 39	M, M	2	Available	+*/Negative
6	USA	31	M	1	None	+
7	Spain	74	F	1	Available	Negative
8	USA	39	F	1	None	+
9	UK	66	M	1	Available	Negative
10	USA	43	M	1	Available	N/A
11	Iran	30	F	1	Available	N/A
12	UK	63	M	1	Available	N/A
13	Germany	61	M	1	Available	Negative
14	USA	26	M	1	None	N/A
15	USA	36	M	1	Available	Negative
16	Indian	22	M	1	Available	N/A
17	Spain	11	F	1	Available	N/A
18	USA	50	M	1	Available	Negative
19	USA	42	M	1	Available	N/A
20	USA	36	M	1	Available	Negative
21	USA	54	M	1	Available	N/A
22	Italy	55	M	1	Available	Negative
23	Spain	55	M	1	None	+

Antecedent infections such as upper respiratory tract infection or gastroenteritis are responsible for the pathogenesis of molecular mimicry [8]. Recently, MFS cases related to vaccines developed against COVID-19 have also started to be reported [9]. There was no vaccination history in our case.

**Figure 1:**
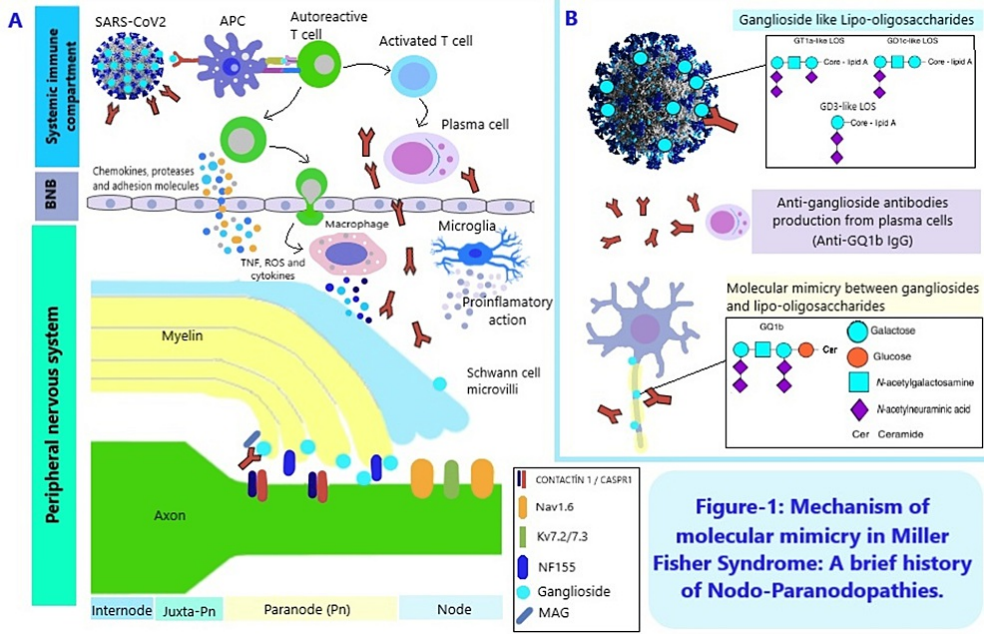
Mechanism of molecular mimicry in MFS: a brief history of nodo-paranodopathies. Panel A and Panel B show that pathophysiological mechanism of COVID-19 associated with MFS. MFS can be triggered by SARS-CoV-2. Myelinated axons are divided into four functional regions: the nodes of Ranvier, paranodes, juxtaparanodes, and internodes. Gangliosides GQ1 and GT1a are strongly expressed at the nodes of Ranvier, where the voltage-gated sodium (Nav) channels are localized. Contactin associated protein (Caspr) and voltage-gated potassium (Kv) channels are respectively present at the paranodes and juxtaparanodes. The pathophysiology of MFS is hypothetically explained by the molecular mimicry mechanism where the virus shares common epitopes in neuron components such as myelin sheath and axon. Invasion of the virus into CNS triggers the activation of the immune system and antigen-presenting precursor cells (APC) that differentiate into T and B lymphocytes. In addition, the viral epitope is recognized by the B lymphocyte and macrophage activation triggers exacerbated local inflammation [Interleukin 4 (IL-4), reactive oxygen species (ROS), and interferon-gamma (IFN-γ)]. Thus, there is the production of antibodies (antiGQ1b) to attack the virus, but these antibodies also attack components of the neuron by sharing epitopes. Autoantibodies developed against the ganglioside-like epitope in the viral lipooligosaccharide structure, as a result of the disruption of the endothelial structure of the BNB due to this inflammatory response, reach and bind to the glycolipids in the GQ-1b structure, especially in the paranodal regions of the nerve. Various proteins are found in the nodal and paranodal regions and have various functions, such as axon-Schwann cell or axon-myelin binding [such as contactin-associated protein 1 (CASPR1), neurofascin 155 (NF155), or contactin 1], sodium channel (NaV). Clustering (like gliomedin) and master node organization. MAG, myelin-associated glycoprotein (Figure adapted from Ref. 12). MFS, Miller Fisher syndrome; BNB, blood nerve barrier

The MFS is a node-paranodapathy. Therefore, it is not myelopathy. Molecular mimicry is the basic pathogenetic mechanism. Molecular mimicry between peripheral nerve and microbial/viral antigens is thought to occur through the activation of the adaptive immune system. Although molecular mimicry is the dominant pathomechanism, recent studies have demonstrated the presence of the neuromuscular transmission defect associated with antiGQ1b antibody formation both in vitro and in vivo. AntiGQ1b antibodies, which act against GQ1b (a ganglioside component of nerves), block acetylcholine release from the motor nerve terminals. It has been reported that antiGQ1b antibody may be positive and that CSF protein can be detected high in different series and case reports, especially in the early stages of the disease, but there are also serogroups with lower antibody positivity. AntiGQ1b antibodies are detected as positive in approximately '85-90,' especially in the MFS variant with ophthalmoplegia and GBS. Even though positivity can be seen in the MFS variant without ophthalmoplegia, positivity is not detected in GBS without ophthalmoplegia [10]. Here, the distribution of ganglioside epitopes in the paranodal regions of the fourth and sixth cranial nerves, mainly in the oculomotor nerves, is seen as a determinant. Considering this information, it is better understood why 85% of the positivity of antiGQ1b antibody can be seen in MFS cases in which external ophthalmoplegic involvement is the anterior plasma. AntiGQ1b antibodies produced against the ganglioside epitopes present here cause conduction block by blocking impulse generation in the nodes of Ranvier [11] (Figure 1).

A review of 123 patients with MFS found that 85% were positive for antiGQ1b [12]. However, a systematic review in 2021 found that a majority of COVID-19-associated MFS cases had negative antiGQ1b results. There is an additional possibility that the positive antiGQ1b results came from non-COVID-19 infections. The antiGQ1b association and the exact pathophysiology of COVID-19 infection leading to the demyelination of the peripheral nervous system are still unclear and warrant further research.

In our case, the dominant findings were areflexia and ataxia with ophthalmoparesis. In the literature, atypical clinical features were observed in antiGQ1b antibody-negative cases. The majority of antibody-negative MFS cases were male, and the disease was observed at an earlier age [13]. Our case was also male, and he had an illness at an early age. Onset is rarely seen with diplopia, but it has been reported that, in the vast majority of antibody-negative cases in large case series, gastroenteritis precedes the clinical table [14]. Contrary to the literature, our case started with diplopia. It should be noted that antibodies such as antiGT1a, GD1b, antiGD2, and GD3 may be positive in antiGQ1b antibody-negative cases [15].

Electroneuromyography (ENMG) studies on MFS show various findings. The most consistent electrophysiological findings in MFS are reduced sensory nerve action potentials (SNAPs) amplitude and absent H reflexes. F wave latencies, known as late responses, and different results regarding H reflexes are reported, but the most common ENMG finding is the absence of H reflexes [16]. F-wave latencies have been reported to be normal, usually prolonged or absent. In our case, F-waves have not obtained both tibials. H reflexes could not be obtained in soleus muscle recordings.

Brain MRI was normal in almost all MFS cases, however, in some case reports, T2 hyperintense images were seen in the cerebral cranial nerves and posterior columns of the spinal cord, especially in the brain [17]. Our case's brain MRI examination was normal in the literature. The main differential diagnosis is Bickerstaff Brainstem Encephalitis (BBSE), which is another cause of post-infectious ophthalmoplegia, ataxia, and areflexia. Protein levels may be elevated in BOS and antiGQ1b antibody may be positive [15]. BBSE was excluded with the presence of areflexia, the absence of pathological reflex, and no disturbance in consciousness.

Molecular mimicry is the main pathogenetic mechanism that causes these trials to be tried in patients with MFS [18]. Immunomodulatory treatments were included in the treatment of MFS with the demonstration of significant curative effects after PE and IV immunoglobulin (IVIg) [18]. However, patients with MFS usually do not require immunotherapy, presumably because they have a good prognosis and spontaneous recovery. Theoretically, circulatory removal or neutralization of pathogenic antibodies should be beneficial in immune-mediated diseases.

The IVIg treatment is generally not necessary in MFS except for BBSE or overlapping GBS because it is costly and does not change the clinical outcome of patients. Although our patient had clinical findings belonging to classical triads, IVIG or PE therapy was not administered for the reason that BBSE or overlapping GBS findings were not present.

## Conclusions

Miller Fisher syndrome is a rare clinical entity that can be seen at any age and can be self-limited. Spontaneous remission is common, and it can be seen more frequently in men at early ages. The clinical course occurring with ophthalmoplegia and ataxia is expanding the spectrum of differential diagnosis. Multiple recurrence episodes can also be observed, usually uniform. It is important to differentiate between the disease BBSE and GBS, which can be diagnosed clinically, immunologically, radiologically, and neurophysiologically. This case, reported as COVID-19-associated MFS, is six years old, the youngest reported in the literature, and the third child case reported after seven and 11 years old child. So far, there have been 25 patients (including our case) reported with MFS associated with COVID-19. Although molecular mimicry is thought to be primarily responsible, the fact that the positivity of an antibody with high specificity and sensitivity such as antiGQ1b is lower in these cases makes it difficult to reach a conclusion about whether the post-infective and/or parainfective pathogenic mechanism is fully effective. Overall, the observation of classical triad clinical findings in COVID-19-associated MFS cases suggests that SARS-CoV-2 directly induces a neuropathogenic effect due to the widespread expression of ACE2 receptors in the nervous system. The antiGQ1b antibody is important but can be detected negatively in most cases of MFS associated with COVID-19. The presence of ACD in CSF with EMG examination seems to have a higher diagnostic value for COVID-19-associated MFS. It should be known that the neurological complications of COVID-19 may start quite early or may occur after a long time, and therefore it should be considered that it would be useful to follow up the clinical conditions with periodic controls. It should be kept in mind that clinical manifestations such as GBS and MFS may also develop due to both COVID-19 itself and vaccines developed against COVID-19.
